# Comparing efficacy of drug-coated balloon-only approach and stent approach in treating de novo coronary artery lesions: A meta-analysis of randomized controlled trials. A protocol for systematic review and meta-analysis

**DOI:** 10.1097/MD.0000000000021295

**Published:** 2020-07-24

**Authors:** Deshuai Yu, Junjun Cai, Kai Wang, Tianli Li, Leilei Liu, Lei Shi, Xian Wang

**Affiliations:** a Dongzhimen Hospital, Beijing University of Chinese Medicine, Beijing; b Department of Hepatology, Tianjin Third Central Hospital, Tianjin; c School of Traditional Chinese Medicine; d School of Life Science; e Institute for Cardiovascular Disease, Beijing University of Chinese Medicine, Beijing, People's Republic of China.

**Keywords:** coronary artery disease, de novo lesion, drug-coated balloon, meta-analysis

## Abstract

**Background:**

Drug-coated balloons (DCB) have been a novel alternative therapeutic strategy in de novo coronary artery diseases. However, the clinical feasibility of the DCB-only approach in treating small vessel disease remains controversial, while study aimed to assess the efficacy and safety of the DCB-only approach versus stent approaches in treating large vessel disease is limited.

**Methods:**

From February 2020 to May 2020, we will search Cochrane Library, PubMed, EMBASE, ScienceDirect, Scopus, Chinese Biomedical Literature Database, Chinese National Knowledge Infrastructure (CNKI), Wanfang Database, and Chongqing VIP Database for eligible trials comparing DCB with drug-eluting stents for treatment of de novo lesions in both small vessel disease and large vessel disease. The primary endpoint is major adverse cardiac events (MACE); the secondary endpoints include in-lesion late lumen loss, binary restenosis, myocardial infarction, target lesion revascularization (TLR), mortality and target vessel thrombosis. Meta-analysis will be conducted using Review Manager software (V.5.3).

**Results:**

The results will be presented as risk ratios for dichotomous data, and weighted mean differences for continuous data.

**Conclusion:**

We will assess outcomes of the DCB-only approach in the treatment of de novo lesions compared with the stent approach.

**PROSPERO registration number:**

CRD42020164484.

## 1 Introduction

At present, metallic stents, especially the drug-eluting stents (DES) are the standard treatment for percutaneous coronary intervention (PCI) in de novo coronary artery disease (CAD), wherein culprit lesions have not previously been treated with angioplasty or stenting. Despite significant advancements that have been made in stent technique, stent implantation can still result in some complex complications, including restenosis, high bleeding risk related to long-term double anti-platelet therapy,^[1]^ late in-stent thrombosis,^[2]^ allergic to stent materials or even stent fracture.^[3]^ Therefore, a suitable alternative for the stent-free interventional treatment of de novo CAD seems to be very promising.

Drug-coated balloons (DCB) are an established therapy for in-stent restenosis (ISR) in bare metal stents (BMS) and DES.^[4–6]^ over the last decade, DCB has been introduced as a potential stand-alone treatment for de novo coronary lesions.^[7–9]^ Although some pilot studies^[8,10]^ showed inferior results for DCBs compared with DES, numerous recently published trials^[11–17]^ have reported that the DCB-only approach for de novo lesions in small coronary arteries is correlated with low rates of major adverse cardiac events (MACE) and target lesion revascularization (TLR). Nevertheless, there was limited research aimed to evaluate the safety and efficacy of the DCB-only strategy versus DES strategy for this indication.^[16,18]^ To clarify this issue, the clinical trial BASKET-SMALL 2^[16]^ compared the paclitaxel-iopromide coated balloon with the second-generation DES in native small coronary lesions, and showed non-inferiority in terms of MACE. Furthermore, a meta-analysis^[19]^ included a total of 5 articles with 1619 patients (1714 lesions) from 2010 to 2018 and suggested that the DCB angioplasty in patients with small sized de novo lesions had a similar risk of TLR and MACE in comparison with DES.

However, most of the above-mentioned studies focused on interventions in small coronary vessels with diameters <2.8 mm (or <3.0 mm), which only constitute about 35% of all the coronary catheter-based procedures.^[20]^ And several recently published clinical trials^[21,22]^ have claimed that, for patients who received DCB as a stand-alone treatment for de novo lesions, the large vessel disease (LVD) group has comparable rates of MACE and TLR than the small vessel disease (SVD) group, suggesting that the DCB-only approach of de novo lesions in large coronary arteries may be as safe and effective as that in small coronary arteries.

In this study, we will carefully summarize current evidence and perform a meta-analysis of randomized controlled trials (RCT), to assess the safety and efficacy of the DCB-only approach versus stent approach of de novo lesions in both large and small coronary vessels.

## 2 Methods

### 2.1 Study registration

We have registered the protocol of this study on the International Prospective Register of Systematic Reviews (registration no. CRD42020164484, which is available on https://www.crd.york.ac.uk/prospero/display_record.php?ID=CRD42020164484), and will conduct this study in accordance with the preferred reporting items for systematic reviews and meta-analyses (PRISMA) statement.^[23]^ No special ethical permission is required for this study, since it is a secondary literature study of published randomized controlled trials.

### 2.2 Data sources and search strategy

#### 2.2.1 Data sources

The electronic databases include: Cochrane Library; PubMed; EMBASE; ScienceDirect; Scopus; Chinese Biomedical Literature Database; Chinese National Knowledge Infrastructure (CNKI); Wanfang Database and Chongqing VIP Database. Reference lists of the selected studies, relevant reviews in the field, and Clinical trial registries (ClinicalTrials.gov and Current Controlled Trials [controlled-trials.com] registries) will also be scrutinized to identify any further studies, but only published literature will be included.

#### 2.2.2 Search strategy

Published studies comparing the efficacy and safety of the DCB-only approach with the stent approach in treating de novo coronary artery lesions will be retrieved in electronic databases and other data sources from February 2020 to May 2020. The search strategy is based on combinations of keywords related to drug-coated balloons (drug-coated balloon, drug-eluting balloon, DCB, DEB, paclitaxel-coated balloon, paclitaxel-eluting balloon, PCB, PEB, sirolimus-coated balloon, sirolimus -eluting balloon, SCB, SEB) and CAD (coronary artery disease, coronary arteriosclerosis). And publication dates for included literatures are restricted to the period from the database construction to May 2020.

### 2.3 Eligibility criteria

#### 2.3.1 Type of studies

Randomized controlled trials published in English or Chinese will be included, while other types of studies (non-RCT design, quasi-experiment design, unobtainable data, duplicate publications, animal experiments, and reviews or case reports) would be excluded.

#### 2.3.2 Type of participants

Eligible patients are:

1. ≥18 years old with de novo CAD demonstrated by coronary angiography ;
2. diagnosed with stable or unstable angina or documented silent ischemia or acute coronary syndrome or MI;
3. treated with a DCB-only approach or a stent approach (BMS or DES) of de novo coronary lesions;
4. receiving standard drug treatments and interventional procedures.

Besides, we will only consider studies that report the patients’ reference vessel diameters. Patients will be further stratified into SVD group and LVD group according to their reference vessel diameters. Since there is no generally accepted definition, we will choose a cut-off of 2.8 mm to distinguish between large and small vessels.

Patients younger than 18 years of age and patients diagnosed with in-stent restenosis would be excluded from this study.

#### 2.3.3 Type of interventions

Studies that compare the DCB-only approach to the BMS or DES approach will be included. The DCB-only approach is defined as using DCB as a stand-alone therapy for de novo lesion after adequate lesion preparation. In the DCB-only arm, stenting is allowed only as a bailout strategy in case of suboptimal results, that is, flow-limiting dissection, persistent residual stenosis, and vessel recoil. Patients who plan to receive stents in combination with DCB therapy would be excluded in this study.

#### 2.3.4 Type of outcome measurements

##### 2.3.4.1 Primary outcomes

The primary endpoint is major adverse cardiac events (MACE), defined as the composite of ischemia-driven TLR, mortality, MI and target vessel thrombosis. All subjects should be followed up for at least 6 months.

##### 2.3.4.2 Secondary outcomes

The secondary endpoints include in-lesion late lumen loss (LLL), binary restenosis (BR), and the components of MACE (TLR, mortality, MI, and target vessel thrombosis). LLL is calculated as the difference in minimal lumen diameter between post-procedure and follow-up, while BR is defined as diameter stenosis ≥50% within a previously treated segment by quantitative coronary angiography at the follow-up angiogram. All subjects should be followed up for at least 6 months.

In the current study, clinical endpoints are considered safety endpoints, including MACE and each of its components (except TLR), whereas TLR and angiographic endpoints (LLL and BR) are considered efficacy endpoints.

### 2.4 Study selection

The results of titles and abstracts of all relevant studies will be merged into the Endnote software, and the duplicates will be removed. Two independent investigators (DY and KW) will screen the titles and abstracts of all citations to identify potentially relevant trials, followed by a review of the full text to assess if trials meet the inclusion criteria. Conflicting findings will be resolved by consensus with a third investigator (JC).

### 2.5 Data extraction

Two independent investigators (JC and DY) will extract information using a predetermined collection form which includes: study design characteristics, selection criteria, relevant population demographics and lesion characteristics, therapeutic measurements, length of follow-up, clinical and angiographic outcomes of interest. Outcomes of interest will be extracted at the longest available follow-up time according to the clinical trial designs. Disagreements will be settled by consensus with a third investigator (KW).

### 2.6 Quality assessment

Methodological qualities of the included studies will be assessed by 2 independent investigators (JC and DY) using the Cochrane Collaboration Risk of Bias tool, which based on the following 7 points: sequence generation, allocation concealment, blinding of participants and personnel, blinding of outcome assessment, incomplete outcome data, selective outcome reporting, and “other issues.” A study will be judged using the labels “low risk,” “high risk,” or “unclear risk.”

### 2.7 Data synthesis and statistical analysis

Statistical analysis will be conducted using Review Manager 5.3 software (Cochrane Collaboration, The Nordic Cochrane Centre, The Cochrane Collaboration, Copenhagen, Denmark). Outcomes analyses will be conducted using frequencies for dichotomous variables and weighted mean differences (MD) for continuous variables. For the effect indicators, Risk ratios (RRs) with 95% confidence intervals (CIs) will be calculated by the Mantel–Haenszel method for dichotomous variables, and weighted mean differences (MDs) with 95% confidence intervals (CIs) will be built with the inverse variance method^[24]^ for continuous variables. *P* < .05 is considered statistically significant for the overall effect.

#### 2.7.1 Assessment of heterogeneity

The heterogeneity of the included studies will be assessed by I^2^ statistics, with I^2^ statistic values <25%, 25% to 50%, and >50% considered as low, moderate, and high levels of heterogeneity, respectively.^[25]^ A standard fixed-effects model (Mantel–Haenszel method) will be applied if the index of heterogeneity (I^2^) is <50%; otherwise, we will choose the Der-Simonian and Laird random-effects model.^[26,27]^

#### 2.7.2 Subgroup analysis

If necessary, analyses will be conducted for overall coronary artery lesions and then stratified by the method of treatments, types of stents in the control group, and calibers of target vessels, or based on other factors that may lead to heterogeneity.

#### 2.7.3 Sensitivity analysis

Sensitivity analysis will be performed by examining the effect of excluding each study.

#### 2.7.4 Assessment of publication biases

Funnel plots and Egger tests will be used to evaluate the potential effects of publication bias, with a *P* value <.05 is considered statistically significant.

## 3 Discussion

Drug-coated balloons are a novel treatment option for de novo CAD that can inhibit neointimal hyperplasia after angioplasty by delivering antiproliferative drugs to the coronary vessel wall without permanently scaffolding. Most of the currently available DCBs are coated with paclitaxel and its derivatives as the antiproliferative drugs, while sirolimus and its derivatives have recently been investigated for DCB applications on the basis of their successes in stent technology.^[28,29]^ However, the vascular response after DCB therapy is influenced not only by the type of antiproliferative drugs, but also by the interaction among the doses, formulations, and release kinetics of the drugs used,^[30]^ which means there is no such thing as a “class effect” of DCBs. Additionally, differences in the study designs, types of stents, demographic and procedural characteristics (ie, the rates of patients with diabetes mellitus, the rates of complicated lesions, the rates of optimal lesion preparation and bailout stenting) among included trails may lead to a relatively high level of heterogeneity in such a meta-analysis that compared the efficacy and safety of DCB-only approach with stent approach. Considering the potential for high heterogeneity, well-designed inclusion criteria, appropriate subgroup analyses, and even meta-regressions are of vital importance for the present study.

## Author contributions

**Conceptualization:** Deshuai Yu, Junjun Cai, Kai Wang.

**Data curation:** Deshuai Yu, Junjun Cai.

**Formal analysis:** Deshuai Yu, Kai Wang.

**Funding acquisition:** Xian Wang.

**Investigation:** Kai Wang, Lei Shi.

**Methodology:** Leilei Liu, Lei Shi.

**Resources:** Deshuai Yu, Junjun Cai.

**Software:** Kai Wang, Tianli Li.

**Supervision:** Xian Wang.

**Writing – original draft:** Deshuai Yu, Junjun Cai.

**Writing – review & editing:** Kai Wang, Xian Wang.
